# Relationship between pulmonary function and peripheral vascular function in older Chinese: Guangzhou biobank cohort study-CVD

**DOI:** 10.1186/s12890-018-0649-x

**Published:** 2018-05-21

**Authors:** Jing Pan, Lin Xu, Tai Hing Lam, Chao Qiang Jiang, Wei Sen Zhang, Feng Zhu, Ya Li Jin, G. Neil Thomas, Kar Keung Cheng, Peymane Adab

**Affiliations:** 1 Guangzhou No.12 Hospital, Guangzhou, Guangdong China; 2 School of Public Health, Sun Yat-sen University, Guangzhou, Guangdong China; 3 School of Public Health, The University of Hong Kong, Hong Kong, China; 4 Institute of Applied Health Research, University of Birmingham, Birmingham, UK

**Keywords:** Pulmonary function, Arterial stiffness, Peripheral arterial disease (PAD), Vascular function

## Abstract

**Background:**

Findings describing the relationship between pulmonary function and peripheral vascular function have been inconclusive. We explored this relationship in Guangzhou Biobank Cohort Study-Cardiovascular Subcohort (GBCS-CVD).

**Methods:**

Brachial-ankle pulse wave velocity (baPWV) and ankle brachial index (ABI) were measured by a waveform analyser, and pulmonary function by turbine flowmeter spirometry. Predicted forced expiratory volume in 1 s (FEV_1_) and forced vital capacity (FVC) were derived using equations for Chinese. Regression analyses were used to investigate the association.

**Results:**

Of 1528 older Chinese, 980 (64.1%) had arterial stiffness (baPWV ≥1400 cm/s), but only 29 (1.9%) had peripheral arterial disease (PAD) (ABI < 0.9). The mean (±standard deviation, SD) baPWV was 1547 (±298) cm/s and mean (±SD) ABI 1.09 (±0.09). Before and after adjusting for potential confounders, baPWV was negatively associated with FEV_1_ and FVC % predicted (% predicted = observed/predicted × 100%) (adjusted β: − 0.95 and − 1.16 respectively, *p* < 0.05), and ABI was marginally non-significantly positively associated with FEV_1_% predicted (adjusted β 0.02, *p* = 0.32) and FVC% predicted (adjusted β 0.02, *p* = 0.18). Compared to participants in the highest tertile of pulmonary function, those in the lowest had higher risk of arterial stiffness (adjusted odds ratio (AOR) 1.51, 95% CI 1.09–2.10 for FEV_1_ and AOR 1.69, 95% CI 1.22–2.33 for FVC), but the higher risk of PAD was marginally non-significant (AOR 1.64, *p* = 0.42 for FEV_1_ and AOR 1.65, *p* = 0.24 for FVC).

**Conclusion:**

In older relatively healthy normal weight Chinese, pulmonary function was inversely dose-dependently associated with arterial stiffness, while the association with PAD was much weaker.

## Background

Cardiovascular disease (CVD), a leading cause of disability and mortality worldwide, has been increasing rapidly in China [1]. Poorer pulmonary function has been reported to be a risk factor for CVD mortality in general population [2], but the mechanisms are unclear. A possible mechanism may be through increasing arterial stiffness, as indicated by elevated pulse wave velocity (PWV) or vascular plaque formation, as indicated by reduced ankle brachial index (ABI). The prevalence of peripheral arterial disease (PAD) (ABI < 0.9) was higher in patients with chronic obstructive pulmonary disease (COPD) (*n* = 2088) than in age- and sex-matched control subjects without COPD (*n* = 4420) (8.8% vs. 1.8%) in the German COPD and Systemic Consequences-Comorbidities Network (COSYCONET) study [3]. Previous studies describing the association between pulmonary function and vascular dysfunction were inconclusive. Many [4–10], but not all [11, 12], of the studies showed that reduced pulmonary function was associated with a higher risk of vascular dysfunction. The discrepancy could be due to interaction, diffrences in study design and, study population (patients or relatively healthy subjects), sample size, and variation in confounding factors considered [5, 7, 8, 10–16].

Peripheral vascular dysfunction has been reported to be influenced by many factors including adiposity [17], blood pressure [18], age [18], physical activity [19] and inflammatory biomarkers such as C-reactive protein [20], which are also associated with pulmonary function. Thus the association between vascular dysfunction and pulmonary function could be influenced by, or depend on these factors. Most of the studies adjusted for age, sex and body mass index (BMI) [4–6, 8, 10–14], but did not adjust for blood pressure, smoking status, physical activity, inflammatory biomarkers and lipids [4, 9, 10]. Although the association did not attenuate substantially after confounder adjustment in most studies, two reported no association after adjusting for sex, age, BMI, total cholesterol/high-density lipoprotein-cholesterol ratio, hypertension, diabetes, drinking and smoking in 678 adult Japanese Americans [11], and after adjusting for sex, age, smoking, highly sensitive C-reactive protein, glucose concentrations, cholesterol-high-density lipoprotein ratio, pulse oximetry oxygen saturation and emphysema severity in 177 patients with chronic obstructive pulmonary disease (COPD) [12]. Only three studies examined whether the association varied by smoking status [4, 21] or sex [14, 21].

Moreover, most of these studies were from Western populations. We found only three studies on the relationship between pulmonary function and vascular function in Asian populations [4, 5, 22] and of them, one was a small hospital-based study (*n* = 155) in China showing a negative association between baPWV and forced expiratory volume in 1 s (FEV_1_) and FEV_1_/forced vital capacity (FVC) ratio after adjusting for age, sex, BMI and smoking status [5]. The other 2 studies were in Japanese populations: a clinical study with only 45 hypertensive patients showing cardio-ankle vascular index (CAVI) was negatively associated with lung function [22], and the other a population-based study (*n* = 8790) which showed a negative association between airflow limitation and baPWV only in smokers [4]. We studied the association between pulmonary function and peripheral vascular function in older Chinese in the Guangzhou Biobank Cohort Study-CVD Sub-study.

## Methods

### Subjects

The Guangzhou Biobank Cohort Study (GBCS), a three-way collaboration among the Guangzhou Number 12 Hospital, the Universities of Hong Kong and Birmingham, recruited 30,518 older Chinese in Guangzhou at baseline from 2003 to 2008 [23]. The Cardiovascular Disease Sub-cohort (GBCS-CVD) included 1996 participants from phase 3 and details have been reported elsewhere [24]. Trained interviewers collected information on the demographic characteristics, family and personal disease and medication history and lifestyle, including smoking, alcohol drinking and physical activity according to the International Physical Activity Questionnaire (IPAQ) using a standardized computer-based questionnaire. Physical examination included height, weight, waist circumference and blood pressure. Blood glucose, lipids and high-sensitivity C-reactive protein (hs-CRP) were assayed after an overnight (> 8 h) fast. Hypertension was defined as systolic blood pressure (SBP) ≥140 mmHg, diastolic blood pressure (DBP) ≥90 mmHg, or self-reported use of antihypertensive medication. Diabetes mellitus (DM) was defined as fasting glucose ≥7.0 mmol/L and/or self-reported DM. Asthma was based on self report of whether a doctor had ever diagnosed them with asthma. Ethical approval was granted by the Guangzhou Medical Ethics Committee of the Chinese Medical Association. All participants gave written, informed consent before participating.

### Exposure indicators

Spirometry was done by a turbine flowmeter (Cosmed microQuark, Rome, Italy), and details of the methods and results on other research questions have been reported elsewhere [25, 26]. Briefly, the pulmonary function test was conducted in a standing position following standard procedures, with at least three maneuvers, and the best measure of FEV_1_ and FVC were recorded. Predicted values for FEV_1_ and FVC were derived using the equations of Ip and colleagues for Chinese [27]. The cutoff points of the tertiles of the FEV_1_% predicted (% predicted = observed/predicted × 100%) were as follows: tertile 1, > 103.4%; tertile 2, 90.5–103.4%; and tertile 3 < 90.5%. The cutoff points of the tertiles of FVC% predicted were as follows: tertile 1 > 102.2%; tertile 2, 89.9–102.2%; and tertile 3, < 89.9%. These cutoff points have been used in our previous paper [26]. We defined COPD based on the presence of airflow obstruction, using the GOLD definition of FEV1/FVC < 0.70. Restrictive pattern of lung disease was defined as FEV1/FVC ≥0.70, FVC% predicted < 0.80.

### Study outcomes

Details of the methods for measuring baPWV and ABI with other results have been reported [24, 28, 29]. Briefly, they were measured in the supine position after 5 min of rest using an automatic waveform analyser (BP-203RPE; Colin Medical Technology, Komaki, Japan), an automated recording device that calculated the time delay between 2 pulse waves recorded simultaneously [24]. Data of the waveforms of both ankles and brachia were stored, the time interval between the wave front of the brachial waveforms and the waveforms of the ankles was automatically measured, which was defined as ΔT. The distance of each segment (La − Lb) was automatically calculated based on the patient’s height. Afterward, baPWV was calculated using the following equation: baPWV (in centimetres per second) = (La − Lb)/ΔT. The mean of the left and right baPWV (average baPWV) was obtained from all subjects and used in the analysis, and the max of the left and right baPWV was also used for sensitivity analysis. Ankle SBP and brachial SBP were measured and ABI was calculated using the equation: ABI = ankle SBP/brachial SBP. The lowest value of the left and right ABI was used for analysis. Higher baPWV (indicating greater arterial stiffness) was defined as baPWV ≥1400 cm/s [30], and lower ABI (indicating peripheral arterial disease (PAD)) as ABI < 0.9 [31].

### Statistical analysis

All data analysis was performed using Stata/SE V.12.0 (StataCorp LP, 4905 Lakeway Drive, College Station, TX77845 USA). Continuous variables were analysed using independent sample t-test and categorical variables using χ^2^ test. Multivariable linear regression was used to calculate regression coefficient (β) and 95% confidence interval (CI) of pulmonary function indicators with vascular function. Multivariable logistic regression was used to calculate odds ratio (OR) of presence of arterial stiffness, and presence of PAD for (a) FEV_1_% predicted tertiles, and (b) FVC% predicted tertiles with and without adjustment for following potential confounders: age (years), sex, waist circumference (cm), education (primary or below, middle school, college or above), smoking (never, former, current smokers (0–29 pack-years) and current smokers (> 30 pack-years)), IPAQ physical activity (physically active, moderate and inactive), diastolic blood pressure (mmHg), triglycerides, fasting plasma glucose (mmol/l), COPD, asthma and medications (aspirin and/or lipid lowering medication).

We tested for interaction between pulmonary function indicators and sex, and smoking status. As we found no evidence that the association of pulmonary function with vascular dysfunction (arterial stiffness or PAD) varied by sex (*P* values for interaction: 0.19–0.99) or smoking status (P values for interaction: 0.15–0.69), we conducted all analysis pooling men and women together with adjustment for sex and smoking. We also performed sensitivity analysis examining the association of pulmonary function indicators with vascular dysfunction by sex and by smoking status. All significance tests were 2-tailed, with *p* < 0.05 as statistically significant.

## Results

Of 1996 participants in GBCS-CVD, 1528 (76.6%) participants with valid data were included in this analysis. Their mean age was 59.3 years (standard deviation, 6.9). Half of the participants were women (50.7%), 64.1% had arterial stiffness, and 1.9% had PAD. 133 (9.2%) participants had obstructive COPD and 154 (10.1%) had restrictive respiratory pattern. Table 1 shows that compared with participants without COPD, those with COPD were older, had lower education and more smokers. They also had lower BMI, cholesterol and pulmonary function, higher blood pressure and baPWV, and higher prevalence of arterial stiffness and PAD, but the differences of history of asthma (based on reporting a doctor diagnosis of the condition), hypertension and diabetes, medication, physical activity level and ABI were non-significant.

**Table 1:** Characteristics of the study sample

	All	Non-COPD	COPD	*P* value
Number	1528	1395	133	
Age, y	59.3 ± 6.9	58.8 ± 6.6	64.7 ± 7.4	< 0.001
Education, n (%)				
≤ Primary	420 (27.5)	361 (25.9)	59 (44.4)	< 0.001
Middle school	913 (59.7)	855 (61.3)	58 (43.6)	
≥ College	195 (12.8)	179 (12.8)	16 (12.0)	
Smoking, n (%)				
Never	1033 (67.6)	998 (70.8)	45 (33.8)	< 0.001
Former	230 (15.1)	198 (14.2)	32 (24.1)	
Current (0–29 pack-years)	127 (8.3)	107 (7.67)	20 (15.0)	
Current (≥ 30 pack-years)	138 (9.0)	102 (7.3)	36 (27.1)	
IPAQ Physical activity, n (%)				
High	917 (60.1)	845 (60.6)	72 (54.2)	0.18
Moderate	456 (29.8)	407 (29.2)	49 (36.8)	
Low	155 (10.1)	143 (10.3)	12 (9.0)	
Body mass index, kg/m^2^	23.8 ± 3.1	23.9 ± 3.0	22.7 ± 3.0	< 0.001
Waist circumference, cm	78.5 ± 9.0	78.5 ± 9.0	78.8 ± 8.5	0.76
History of diseases				
Asthma, n (%)	19 (1.2)	19 (1.4)	0 (0.0)	0.18
Hypertension, n (%)	526 (34.4)	471 (33.8)	55 (41.4)	0.08
Diabetes, n (%)	95 (6.2)	84 (6.0)	11 (8.3)	0.31
Systolic blood pressure, mmHg	127 ± 21	127 ± 20	133 ± 23	0.003
Diastolic blood pressure, mmHg	74 ± 11	74 ± 11	75 ± 11	0.16
Total cholesterol, mmol/l	5.82 ± 1.09	5.84 ± 1.09	5.59 ± 1.09	0.01
HDL-cholesterol, mmol/l	1.58 ± 0.40	1.58 ± 0.41	1.59 ± 0.37	0.92
LDL-cholesterol, mmol/l	3.36 ± 0.68	3.38 ± 0.68	3.22 ± 0.67	0.01
Triglycerides, mmol/l	1.83 ± 1.42	1.85 ± 1.46	1.58 ± 0.95	0.04
Fasting plasma glucose, mmol/l	5.59 ± 1.46	5.60 ± 1.51	5.51 ± 0.93	0.48
hs-CRP, mg/l (*n* = 1518)	2.51 ± 2.89	2.45 ± 2.80	3.13 ± 3.66	0.009
hs-CRP, mg/l^a^	1.45 (1.37–1.53)	1.44 (1.34–1.55)	1.46 (1.35–1.58)	0.71
Aspirin, n (%)	66 (4.3)	61 (4.4)	5 (3.8)	0.74
Lipid lowering medication, n (%)	48 (3.1)	45 (3.2)	3 (2.3)	0.54
FEV_1_% predicted, %	96.1 ± 16.6	98.0 ± 14.8	75.7 ± 20.6	< 0.001
FVC% predicted, %	95.9 ± 14.7	96.5 ± 13.9	89.9 ± 20.3	< 0.001
FEV_1_/FVC ratio, %	78.4 ± 6.9	79.8 ± 5.0	63.8 ± 7.2	< 0.001
Mean baPWV, cm/s	1547 ± 298	1535 ± 294	1664 ± 323	< 0.001
Arterial stiffness, n (%)	980 (64.1)	879 (63.0)	101 (75.9)	0.003
ABI	1.09 ± 0.09	1.09 ± 0.09	1.08 ± 0.09	0.34
PAD, n (%)	29 (1.9)	23 (1.7)	6 (4.5)	0.02

Results shown as mean ± standard deviation or number (%)

^a^Results shown as geometric mean and 95% confidence interval

*IPAQ* International Physical Activity Questionnaire, *COPD* chronic obstructive pulmonary disease, *HDL* high density lipoprotein, *LDL* low density lipoprotein, *hs-CRP* high-sensitivity C-reactive protein, *FEV*_*1*_ forced expiratory volume in 1 s, *FVC* forced vital capacity, *baPWV* brachial-ankle pulse wave velocity, *ABI* ankle-brachial index, *PAD* peripheral arterial disease;

COPD: FEV1/FVC < 0.70

Asthma: self-reported doctor-diagnosed asthma

Hypertension: systolic blood pressure (SBP) ≥140 mmHg, diastolic blood pressure ≥ 90 mmHg, or self-reported use of antihypertensive medication

Diabetes: fasting glucose ≥7.0 mmol/L and/or self-reported DM

Arterial stiffness: baPWV≥1400 cm/s

PAD: ABI < 0.9

Table 2 shows that baPWV was significantly inversely associated with all pulmonary function indicators (Crude β ranged from − 3.02 to − 5.87, *p* < 0.001) in simple linear regression models. After adjusting for age, sex, education, smoking, physical activity, waist circumference, diastolic blood pressure, triglycerides, fasting plasma glucose, COPD, asthma and medications (aspirin and/or lipid lowering medication), the inverse association of baPWV with FEV_1_% predicted (adjusted β-0.95, 95% CI -1.70 to − 0.20) and FVC % predicted (adjusted β-1.16, 95% CI -1.98 to − 0.35) remained significant, and that of FEV_1_ /FVC ratio became non-significant (*p* = 0.91). For ABI, significantly positive association was found for FEV_1_% predicted (β = 0.03, *p* = 0.045) but not FVC% predicted (β = 0.03, *p* = 0.06) and FEV_1_/FVC ratio (β = 0.02, *p* = 0.54). After similar adjustment, those associations became non-significant (adjusted β 0.02, 0.02 and − 0.005, all *p* values > 0.05).

**Table 2:** Association (regression coefficient β, 95% confidence interval) of pulmonary function with baPWV and ABI in all participants

	Unadjusted β (95% CI)	P	Adjusted β (95% CI)	P
baPWV (dependent variable)				
FEV_1_% predicted	−3.02 (−3.91 to −2.13)	< 0.001	−0.95 (−1.70 to −0.20)	0.01
FVC% predicted	−4.34 (−5.34 to −3.35)	< 0.001	−1.16 (−1.98 to − 0.35)	0.005
FEV_1_ /FVC ratio	−5.87 (−8.02 to − 3.71)	< 0.001	0.53 (− 1.27 to 2.33)	0.91
ABI (dependent variable)				
FEV_1_% predicted	0.03 (0.001 to 0.05)	0.045	0.02 (−0.01 to 0.04)	0.32
FVC% predicted	0.03 (−0.002 to 0.06)	0.06	0.02 (−0.01 to 0.05)	0.18
FEV_1_ /FVC ratio	0.02 (−0.04 to 0.08)	0.54	−0.005 (− 0.10 to 0.09)	0.92

*baPWV* brachial-ankle pulse wave velocity (greater variables indicate greater stiffness), *ABI* ankle-brachial index (lower variables indicate greater stiffness), *FEV*_*1*_ forced expiratory volume in 1 s, *FVC* forced vital capacity

Adjusted for age, sex, education, smoking, physical activity, waist circumference, diastolic blood pressure, triglycerides, fasting plasma glucose, chronic obstructive pulmonary disease, asthma and medications (aspirin and/or lipid lowering medication)

Compared with participants in the highest tertile, those in the lowest tertile of pulmonary function showed a higher risk of arterial stiffness (adjusted odd ratio (AOR) 1.51, 95% CI 1.09–2.10 for FEV_1_% predicted and AOR 1.69, 95% CI 1.22–2.33 for FVC% predicted) (Table 3 and Table 4). The AOR of lowest tertile of pulmonary function with higher risk of lower ABI was statistically non-significant (AOR 1.64, 95% CI 0.54–5.01 for FEV_1_% predicted and AOR 1.65, 95% CI 0.63–4.29 for FVC% predicted). Poorer pulmonary function showed dose-response relationship with arterial stiffness (*P* values for trend < 0.03), instead of PAD (*P* values for trend > 0.05). Stratified analysis by sex (Appendix 1 Table 5 and (Appendix 2 Table 6) and by smoking status (Appendix 3 Table 7 and Appendix 4 Table 8) showed similar tendencies. Sensitivity analysis of the relationship between pulmonary function and the maximal values of left and right baPWV showed similar results with the means of left and right baPWV (data not shown).

**Table 3:** Adjusted odds ratios (ORs) for the presence of peripheral vascular dysfunction by tertiles of FEV_1_% predicted

	FEV_1_% predicted (%)			P for trend
	Tertile 1	Tertile 2	Tertile 3	
Range, %	> 103.4	90.5–103.4	< 90.5	
Number of participants	509	509	510	
Presence of arterial stiffness, n (%)	293 (57.6)	313 (61.5)	374 (73.3)	< 0.001
Crude OR (95% CI)	1.00	1.18 (0.92–1.51)	2.03 (1.56–2.64)**	< 0.001
Adjusted OR (95% CI)	1.00	1.14 (0.85–1.54)	1.51 (1.09–2.10)*	0.02
Presence of PAD, n (%)	5 (1.0)	10 (2.0)	14 (2.8)	
Crude OR (95% CI)	1.00	2.02 (0.69–5.95)	2.85 (1.02–7.96)*	0.04
Adjusted OR (95% CI)	1.00	1.67 (0.56–4.99)	1.64 (0.54–5.01)	0.42

*FEV*_*1*_ forced expiratory volume in 1 s, *PAD* peripheral arterial disease

Adjusted for age, sex, education, smoking, physical activity, waist circumference, diastolic blood pressure, triglycerides, fasting plasma glucose, chronic obstructive pulmonary disease, asthma and medications (aspirin and/or lipid lowering medication)

Arterial stiffness: brachial-ankle pulse wave velocity (baPWV) ≥1400 cm/s

PAD: ankle-brachial index (ABI) < 0.9

*P < 0.05; **:*P* < 0.01

**Table 4:** Adjusted odds ratios (ORs) for the presence of peripheral vascular dysfunction by tertiles of FVC% predicted

	FVC% predicted (%)			P for trend
	Tertile 1	Tertile 2	Tertile 3	
Range, %	> 102.2	89.9–102.2	< 89.9	
Number of participants	509	509	510	
Presence of arterial stiffness, n (%)	283 (55.6)	312 (61.3)	385 (75.5)	< 0.001
Crude OR (95% CI)	1.00	1.26 (0.99–1.62)	2.46 (1.88–3.21)**	< 0.001
Adjusted OR (95% CI)	1.00	1.10 (0.82–1.49)	1.69 (1.22–2.33)**	0.002
Presence of PAD, n (%)	7 (1.4)	6 (1.2)	16 (3.1)	
Crude OR (95% CI)	1.00	0.86 (0.29–2.56)	2.32 (0.95–5.69)	0.04
Adjusted OR (95% CI)	1.00	0.79 (0.26–2.42)	1.65 (0.63–4.29)	0.24

*FVC* forced vital capacity, *PAD* peripheral arterial disease

Adjusted for age, sex, education, smoking, physical activity, waist circumference, diastolic blood pressure, triglycerides, fasting plasma glucose, chronic obstructive pulmonary disease, asthma and medications (aspirin and/or lipid lowering medication)

Arterial stiffness: brachial-ankle pulse wave velocity (baPWV) ≥1400 cm/s

PAD: ankle-brachial index (ABI) < 0.9

**:P < 0.01

## Discussion

Our study was the first population-based study on older relatively healthy and normal weight Chinese with comprehensive adjustment for many potential confounders showing that pulmonary function was inversely dose-dependently associated with arterial stiffness, while the association with PAD was much weaker. Furthermore, we found no evidence that the association varied by sex or smoking status.

Our study suggests reduced pulmonary function might increase risk of cardiovascular disease through actions on arterial stiffness in general population. Several hospital-based studies showed an inverse association between arterial stiffness and pulmonary function [5, 6, 12, 13, 22] despite small sample size (*n* < 250). The COSYCONET study found that 8.8% of patients with COPD had PAD, compared to only 1.8% in age- and sex-matched control subjects without COPD [3]. Our findings are consistent with a few earlier population-based studies, including the Atherosclerosis Risk in Communities (ARIC) Study (*n* = 14,000) [21], the Nagahama Study (*n* = 8790) [4], the Whitehall II Study (*n* = 5392) [8], the Copenhagen City Heart Study (*n* = 3374) [7], the Caerphilly Prospective Study (*n* = 827) [9], the “Men born in 1914” cohort (*n* = 207) [32] and the Burden of Lung Disease (BOLD) study (*n* = 108) [10], which were predominantly conducted in Caucasian populations. PWV [4, 8–10], ABI [21, 32] and aortic augmentation index (AIx) [14] were used as indicators for vascular function in these studies. ABI indicates PAD, while PWV indicates arterial stiffness. The combination of both should provide more comprehensive information of peripheral vascular function, but none of the former studies explored both indices. Hence our study is the first showing that poorer pulmonary function was associated with arterial stiffness, while the association with PAD was much weaker. Our findings are in line with our former research based on the same participants: pulmonary function was significantly associated with common carotid artery intima-media thickness (IMT), but the association between pulmonary function and carotid plaque was marginally non-significant [26].

The association may result from common risk factors, confounders, or mediators such as age, BMI, physical activity, smoking, hyperglycemia, dyslipidemia and inflammation [4, 8, 11]. Some, such as age, sex, and BMI were adjusted for in most studies [4–6, 8, 10–14], whereas others such as blood pressure, smoking status, physical activity, inflammation and lipids were not, probably because of the lack of data [4, 9, 10]. Most of the studies found that the association could not be explained by the confounding factors [5, 8]. However, in two studies [11, 12], after adjusting for potential confounders, no difference in vascular dysfunction was found with pulmonary function, possibly due to the additional adjustment for blood pressure not performed by other studies, and the relatively small sample size (533 subjects with normal and 145 with abnormal PWV) [11].

Only a few studies conducted subgroup analysis by sex [14] or smoking status [4]. The Copenhagen City Heart Study showed that the association between AIx and FEV_1_ was significant in both men and women [14], which was in concordance with two other studies involving men only [7, 9]. Elevated cardiovascular morbidity after menopause in women might be due to the decreased secretion of estrogen which can protect cardiovascular system [33]. The risks of vascular disease were similar in both sexes in elder populations [34], so were the risks of vascular dysfunction in both sexes in our older Chinese cohort. This might be the explanation for the absence of sex interaction. Smoking was reported as an important risk factor for both vascular disease and poorer pulmonary function. In the Nagahama Study, airflow limitation was associated with baPWV in smokers, but not in non-smokers [4]. But in a relative healthy population, there might be some underlying mechanisms (aging [35, 36], parallel physiological pathways for elastic changes [37] and inflammatory responses [37]) which could have stronger impacts for the association between lower pulmonary function and peripheral vascular dysfunction than smoking. In summary, our results are consistent with the results elsewhere and can add to the literature by showing that poor pulmonary function is independently associated with a higher risk of peripheral vascular dysfunction regardless of sex and smoking in an older Chinese population.

The underlying mechanisms for the findings are not fully understood. There are some possible explanations. Aging is known to influence both vascular (due to the aging-related influence on the nonliving elastic fibers of the arterial wall) [35] and pulmonary function (due to the aging-related decrease in the static elastic recoil of the lung and compliance of the chest wall, and in the strength of respiratory muscles) [36]. However, the present study found independent associations after adjusting for multiple confounders including age. These findings indicate that other mechanisms instead of aging contributed to the associations. Parallel physiological pathways for elastic changes in the vasculature and lung parenchyma tissue might be the link of the parallel declines in arterial elasticity and lung function [12, 37, 38]. Matrix metalloprotease (MMP)-9 and tissue inhibitor of metalloproteinase-1 (TIMP-1) which were reported to be associated with arterial stiffness [39] were higher in patients with emphysema [40] and COPD [41]. Alteration in elastase or MMP due to genetic or proteolytic process might influence both the connective tissue of the alveoli and arterial wall, and reduce their elasticity [37]. Previous studies have shown that arterial stiffness is associated with severity of emphysema, independently of airflow obstruction [12, 38]. This is hypothesised to be due to a common pathophysiological process, with elastin degradation affecting both alveolar and arterial walls [42]. In our cohort, although we were not able to assess emphysema using imaging or other measures, the participants with COPD were almost all lean, consistent with an emphysematous or “pink puffer” phenotype. Another mechanism might be inflammatory responses, which play important roles in both vascular and respiratory system [37]. However, the BOLD study analysed the association of pulmonary function with arterial stiffness and inflammatory biomarkers including C-reactive protein, interleukin 8, tumour necrosis factor alpha, MMP-9, and tissue inhibitor of metalloproteinase 1, but found no significant association even before adjustments of age, sex, height, ethnicity, BMI, smoking status and pack-years [10], suggesting inflammation could not fully explain the results.

There were several limitations in our study. First, because of the cross-sectional design, whether the associations between pulmonary function and peripheral vascular dysfunction are causal could not be ascertained. Second, we did not perform post-bronchodilator spirometry, although all earlier population-based studies were also based on pre-bronchodilator measurements only. In addition, we relied on airway obstruction to define COPD, with no detailed measurements (e.g. CT scan, body plethysmography and diffusion capacity of the lung for carbon monoxide) to differentiate phenotypes. We were therefore not able to examine whether any association differed by phenotype. However, if such measurements would reveal more lung abnormalities in subjects with better pulmonary function, the strength of our observed association between pulmonary function and peripheral vascular function could have been under-estimated. Fourth, some of the confounders could also be mediators, such as BMI and physical activity, and the multivariate adjustment could have led to an underestimation of the true effect size. However, it is impossible to completely distinguish mediating and confounding effects in our analysis. Finally, our sample size might not be sufficient to detect small interaction effects.

## Conclusions

Pulmonary function was inversely dose-dependently associated with arterial stiffness in older relatively healthy and normal weight Chinese individuals after adjusting for multiple potential confounders, while the association with PAD was much weaker. Furthermore, the association did not vary by sex and smoking. This paper might provide more information in the field of cardiovascular-pulmonary interactions. Further intervention studies examining the effect of improving pulmonary function on improving vascular function are warranted. Non-invasive measurements of vascular dysfunction in individuals with poor pulmonary function or vice versa might help to identify those with both conditions and who need special clinical management.

## Appendix 1

**Table 5:** Adjusted odds ratios (ORs) for the presence of peripheral vascular dysfunction by tertiles of FEV_1_% predicted and by sex

	FEV_1_% predicted (%)			P for trend
	Tertile 1	Tertile 2	Tertile 3	
Men				
Range, %	> 101.5	87.0–101.5	< 87.0	
Number of participants	251	251	251	
Presence of arterial stiffness, n (%)	174 (69.3)	181 (72.1)	202 (80.5)	
Crude OR (95% CI)	1.00	1.14 (0.78–1.68)	1.82 (1.21–2.75)**	0.005
Adjusted OR (95% CI)	1.00	1.20 (0.77–1.89)	1.75 (1.05–2.92)*	0.03
Presence of PAD, n (%)	4 (1.6)	5 (2.0)	7 (2.8)	
Crude OR (95% CI)	1.00	1.26 (0.33–4.73)	1.77 (0.51–6.13)	0.36
Adjusted OR (95% CI)	1.00	1.18 (0.31–4.57)	1.61 (0.42–6.16)	0.48
Women				
Range, %	> 105.7	94.1–105.7	< 94.1	
Number of participants	258	258	259	
Presence of arterial stiffness, n (%)	120 (46.5)	150 (58.1)	153 (59.1)	
Crude OR (95% CI)	1.00	1.60 (1.13–2.26)**	1.66 (1.17–2.35)**	0.004
Adjusted OR (95% CI)	1.00	1.50 (0.99–2.27)	1.28 (0.83–1.96)	0.25
Presence of PAD, n (%)	2 (0.8)	3 (1.2)	8 (3.1)	
Crude OR (95% CI)	1.00	1.51 (0.25–9.09)	4.08 (0.86–19.40)	0.05
Adjusted OR (95% CI)	1.00	1.08 (0.17–7.12)	1.62 (0.27–9.67)	0.56

*FEV*_*1*_ forced expiratory volume in 1 s, *PAD* peripheral arterial disease

Adjusted for age, education, smoking, physical activity, waist circumference, diastolic blood pressure, triglycerides, fasting plasma glucose, chronic obstructive pulmonary disease, asthma and medications (aspirin and/or lipid lowering medication)

Arterial stiffness: brachial-ankle pulse wave velocity (baPWV) ≥1400 cm/s

PAD: ankle-brachial index (ABI) < 0.9

P for sex interaction: (1) Arterial stiffness: 0.99; (2) PAD: 0.27

**:P* < 0.05; ***:P* < 0.01

## Appendix 2

**Table 6:** Adjusted odds ratios (ORs) for the presence of peripheral vascular dysfunction by tertiles of FVC% predicted and by sex

	FVC% predicted (%)			P for trend
	Tertile 1	Tertile 2	Tertile 3	
Men				
Range, %	> 100.6	87.3–100.6	< 87.3	
Number of participants	251	251	251	
Presence of arterial stiffness, n (%)	168 (69.3)	181 (72.1)	208 (82.9)	
Crude OR (95% CI)	1.00	1.28 (0.87–1.87)	2.39 (1.57–3.64)**	< 0.001
Adjusted OR (95% CI)	1.00	1.35 (0.86–2.11)	2.18 (1.32–3.59)**	0.002
Presence of PAD, n (%)	4 (1.6)	6 (2.4)	6 (2.4)	
Crude OR (95% CI)	1.00	1.51 (0.42–5.43)	1.51 (0.42–5.43)	0.54
Adjusted OR (95% CI)	1.00	1.46 (0.39–5.44)	1.32 (0.35–5.04)	0.69
Women				
Range, %	> 105.7	94.1–105.7	< 94.1	
Number of participants	258	258	259	
Presence of arterial stiffness, n (%)	118 (45.7)	139 (53.9)	166 (64.1)	
Crude OR (95% CI)	1.00	1.39 (0.98–1.96)	2.12 (1.49–3.01)**	< 0.001
Adjusted OR (95% CI)	1.00	1.14 (0.76–1.72)	1.22 (0.79–1.89)	0.36
Presence of PAD, n (%)	3 (1.2)	3 (1.2)	7 (2.7)	
Crude OR (95% CI)	1.00	1.00 (0.20–5.00)	2.36 (0.60–9.23)	0.18
Adjusted OR (95% CI)	1.00	0.70 (0.12–3.92)	1.18 (0.24–5.87)	0.82

*FVC* forced vital capacity, *PAD* peripheral arterial disease

Adjusted for age, education, smoking, physical activity, waist circumference, diastolic blood pressure, triglycerides, fasting plasma glucose, chronic obstructive pulmonary disease, asthma and medications (aspirin and/or lipid lowering medication)

Arterial stiffness: brachial-ankle pulse wave velocity (baPWV) ≥1400 cm/s

PAD: ankle-brachial index (ABI) < 0.9

P for sex interaction: (1) Arterial stiffness: 0.19; (2) PAD: 0.69

***:P* < 0.01

## Appendix 3

**Table 7:** Adjusted odds ratios (ORs) for the presence of peripheral vascular dysfunction by tertiles of FEV_1_% predicted and by smoking

	FEV_1_% predicted (%)			P for trend
	Tertile 1	Tertile 2	Tertile 3	
Non-smoker				
Range, %	> 104.1	91.4–104.1	< 91.4	
Number of participants	344	344	345	
Presence of arterial stiffness, n (%)	181 (53.6)	211 (61.3)	230 (66.7)	
Crude OR (95% CI)	1.00	1.43 (1.06–1.93)*	1.80 (1.32–2.45)**	< 0.001
Adjusted OR (95% CI)	1.00	1.44 (1.00–2.07)	1.35 (0.93–1.98)	0.10
Presence of PAD, n (%)	2 (0.6)	7 (2.0)	8 (2.3)	
Crude OR (95% CI)	1.00	3.55 (0.73–17.22)	4.06 (0.86–19.26)	0.08
Adjusted OR (95% CI)	1.00	3.41 (0.69–16.86)	3.85 (0.78–18.97)	0.11
Ever-smoker				
Range, %	> 99.4	85.4–99.4	< 85.4	
Number of participants	165	165	165	
Presence of arterial stiffness, n (%)	112 (67.9)	112 (67.9)	134 (81.2)	
Crude OR (95% CI)	1.00	1.00 (0.63–1.59)	2.05 (1.23–3.40)**	0.007
Adjusted OR (95% CI)	1.00	1.13 (0.65–1.96)	2.09 (1.09–4.01)*	0.03
Presence of PAD, n (%)	2 (1.2)	4 (2.4)	6 (3.6)	
Crude OR (95% CI)	1.00	2.02 (0.37–11.21)	3.08 (0.61–15.47)	0.16
Adjusted OR (95% CI)	1.00	2.56 (0.38–17.28)	1.47 (0.21–10.11)	0.81

*FEV*_*1*_ forced expiratory volume in 1 s, *PAD* peripheral arterial disease

Adjusted for age, sex, education, physical activity, waist circumference, diastolic blood pressure, triglycerides, fasting plasma glucose, chronic obstructive pulmonary disease, asthma and medications (aspirin and/or lipid lowering medication)

Arterial stiffness: brachial-ankle pulse wave velocity (baPWV) ≥1400 cm/s

PAD: ankle-brachial index (ABI) < 0.9

P for smoking interaction: (1) Arterial stiffness: 0.50; (2) PAD: 0.69

**:P* < 0.05; ***:P* < 0.01

## Appendix 4

**Table 8:** Adjusted odds ratios (ORs) for the presence of peripheral vascular dysfunction by tertiles of FVC% predicted and by smoking

	FVC% predicted (%)			P for trend
	Tertile 1	Tertile 2	Tertile 3	
Non-smoker				
Range, %	> 102.6	90.8–102.6	< 90.8	
Number of participants	344	344	345	
Presence of arterial stiffness, n (%)	176 (51.2)	204 (59.3)	242 (70.1)	
Crude OR (95% CI)	1.00	1.39 (1.03–1.88)*	2.24 (1.64–3.07)**	< 0.001
Adjusted OR (95% CI)	1.00	1.17 (0.82–1.68)	1.37 (0.94–2.01)	0.10
Presence of PAD, n (%)	4 (1.2)	4 (1.2)	9 (2.6)	
Crude OR (95% CI)	1.00	1.00 (0.25–4.03)	2.23 (0.77–6.49)	0.14
Adjusted OR (95% CI)	1.00	0.91 (0.22–3.78)	2.09 (0.61–7.24)	0.20
Ever-smoker				
Range, %	> 100.0	87.0–100.0	< 87.0	
Number of participants	165	165	165	
Presence of arterial stiffness, n (%)	109 (66.1)	115 (69.7)	134 (81.2)	
Crude OR (95% CI)	1.00	1.18 (0.74–1.88)	2.22 (1.34–3.68)*	0.002
Adjusted OR (95% CI)	1.00	1.07 (0.61–1.87)	1.98 (1.08–3.61)*	0.03
Presence of PAD, n (%)	3 (1.8)	3 (1.8)	6 (3.6)	
Crude OR (95% CI)	1.00	1.00 (0.20–5.03)	2.04 (0.50–8.29)	0.29
Adjusted OR (95% CI)	1.00	1.19 (0.22–6.54)	1.10 (0.23–5.05)	0.91

*FVC* forced vital capacity, *PAD* peripheral arterial disease

Adjusted for age, sex, education, physical activity, waist circumference, diastolic blood pressure, triglycerides, fasting plasma glucose, chronic obstructive pulmonary disease, asthma and medications (aspirin and/or lipid lowering medication)

Arterial stiffness: brachial-ankle pulse wave velocity (baPWV) ≥1400 cm/s

PAD: ankle-brachial index (ABI) < 0.9

P for smoking interaction: (1) Arterial stiffness: 0.15; (2) PAD: 0.54

**:P* < 0.05; **:*P* < 0.01

## Abbreviations

ABI: Ankle brachial index
AIx: Aortic augmentation index
ARIC: Atherosclerosis Risk in Communities
baPWV: Brachial-ankle pulse wave velocity
BMI: Body mass index
BOLD: Burden of Lung Disease
CI: Confidence interval
COPD: Chronic obstructive pulmonary disease
COSYCONET: German COPD and Systemic Consequences-Comorbidities Network cohort
CVD: Cardiovascular Subcohort
DBP: Diastolic blood pressure
DM: Diabetes mellitus
FEV1: Forced expiratory volume in 1 s
FVC: Forced vital capacity
GBCS: Guangzhou Biobank Cohort Study
hs-CRP: High-sensitivity C-reactive protein
IPAQ: International Physical Activity Questionnaire
MMP: Matrix metalloprotease
OR: Odd ratio
PAD: Peripheral artery disease
SBP: Systolic blood pressure
TIMP-1: Tissue inhibitor of metalloproteinase-1
